# Feasibility study of adaptive radiotherapy for esophageal cancer using artificial intelligence autosegmentation based on MR-Linac

**DOI:** 10.3389/fonc.2023.1172135

**Published:** 2023-06-08

**Authors:** Huadong Wang, Xin Liu, Yajun Song, Peijun Yin, Jingmin Zou, Xihua Shi, Yong Yin, Zhenjiang Li

**Affiliations:** ^1^ Department of Graduate, Shandong First Medical University (Shandong Academy of Medical Sciences), Jinan, China; ^2^ Department of Radiation Oncology Physics and Technology, Shandong Cancer Hospital and Institute, Shandong First Medical University and Shandong Academy of Medical Sciences, Jinan, China; ^3^ Department of Clinical Medicine, Southwestern Medical University, Luzhou, China; ^4^ College of Physics and Electronic Science, Shandong Normal University, Jinan, China

**Keywords:** esophageal cancer, adaptive radiotherapy, MR-linac, artificial intelligence, automatic segmentation

## Abstract

**Objective:**

We proposed a scheme for automatic patient-specific segmentation in Magnetic Resonance (MR)-guided online adaptive radiotherapy based on daily updated, small-sample deep learning models to address the time-consuming delineation of the region of interest (ROI) in the adapt-to-shape (ATS) workflow. Additionally, we verified its feasibility in adaptive radiation therapy for esophageal cancer (EC).

**Methods:**

Nine patients with EC who were treated with an MR-Linac were prospectively enrolled. The actual adapt-to-position (ATP) workflow and simulated ATS workflow were performed, the latter of which was embedded with a deep learning autosegmentation (AS) model. The first three treatment fractions of the manual delineations were used as input data to predict the next fraction segmentation, which was modified and then used as training data to update the model daily, forming a cyclic training process. Then, the system was validated in terms of delineation accuracy, time, and dosimetric benefit. Additionally, the air cavity in the esophagus and sternum were added to the ATS workflow (producing ATS+), and the dosimetric variations were assessed.

**Results:**

The mean AS time was 1.40 [1.10–1.78 min]. The Dice similarity coefficient (DSC) of the AS model gradually approached 1; after four training sessions, the DSCs of all ROIs reached a mean value of 0.9 or more. Furthermore, the planning target volume (PTV) of the ATS plan showed a smaller heterogeneity index than that of the ATP plan. Additionally, V5 and V10 in the lungs and heart were greater in the ATS+ group than in the ATS group.

**Conclusion:**

The accuracy and speed of artificial intelligence–based AS in the ATS workflow met the clinical radiation therapy needs of EC. This allowed the ATS workflow to achieve a similar speed to the ATP workflow while maintaining its dosimetric advantage. Fast and precise online ATS treatment ensured an adequate dose to the PTV while reducing the dose to the heart and lungs.

## 1 Introduction

Esophageal cancer (EC) is one of the most common malignancies, with a global incidence of 3.2% (1). It ranks seventh among all malignant tumors in terms of commonality, has a mortality rate of 5.3%, ranking sixth, and has a 5-year survival rate of only 20% (2). For patients who refuse surgery or who have locally advanced EC that cannot be resected, radiotherapy is one of the best treatment options (3, 4).

However, conventional radiation therapy cannot fully address the inherent motion of the esophagus in the intrafraction period and the recontouring of the target area after changes in tumor location and shape in the interfraction period, but the advent of MR-Linac has addressed these issues well (5). The Unity MR-Linac (Elekta AB, Stockholm, Sweden) integrates a 7 MV linear accelerator and a 1.5 T diagnostic MRI scanner (6, 7) and provides both adapt-to-position (ATP) and adapt-to-shape (ATS) workflows. The ATP workflow is only required to register the reference CT and online MRI, correct the plan isocenter locations, and reoptimize the plan. Although the time required for the ATP workflow is short, the accuracy of the dose delivery cannot be guaranteed. For the ATS workflow, it is necessary to redelineate regions of interest (ROIs) to adapt the organ changes online. While improving the precision of dose delivery, the therapy time is greatly prolonged. Therefore, short therapy times and precise dose delivery cannot be achieved simultaneously (8).

In summary, the main task is currently to improve the speed of online ROI delineation in the ATS workflow, as a lower delineation time would reduce the probability of patient displacement and improve the accuracy of the dose. Fortunately, the emergence of automatic artificial intelligence (AI)–based delineation has not only guaranteed better segmentation speeds but also shown better segmentation quality (9). This has laid a solid foundation for the application of AI-based autosegmentation (AS) in MR-guided online adaptive radiotherapy (MRgoART) (10). Unfortunately, its clinical applicability is often limited, mainly because most available automatic segmentation measures are based on the deep learning of large samples (11–13). Additionally, similar high-quality images are not easily collected, and the reproduction of experimental results is difficult. This implies that there are barriers to the application of AI in the ATS workflow (14, 15).

In addition, the ATS plan is based on the average electron density assignment of structures on the reference CT for generating a new plan. Dose accuracy at the treatment site is not significantly reduced when using MRI data given sufficient bulk density (16). Additionally, the bulk density assigned to bone tissue and cavities in particular is often inaccurate (17, 18). Therefore, the dosimetric errors arising from both the sternum and the air cavity in the esophagus during the ATS treatment workflow of EC tend to be unknown.

Therefore, facing the above two problems, the main objective of this study was to propose a scheme for automatic patient-specific segmentation in MRgoART based on daily updated, small-sample deep learning models to address the time-consuming nature of ROI delineation in the ATS workflow. Additionally, we sought to verify the feasibility of the use of this scheme in adaptive radiotherapy for EC in terms of the delineation time, accuracy, and dosimetric benefit. Finally, the dosimetric errors arising from the absence of both the air cavity in the esophagus and sternal structures in the ATS workflow were explored.

## 2 Materials and methods

### 2.1 Patient information

Nine patients with EC who underwent MR-guided online adaptive radiotherapy in our research center between September 2021 and June 2022 were prospectively included in this study. There were a total of 216 treatment fractions, with 72 (8 fractions*9 patients) treatment fractions each for the ATP plan, ATS plan, and ATS+ (the ATS plan augmented with the newly added air cavity in the esophagus and the sternum) plan. **Table 1** summarizes the patient characteristics. This study was approved by the Ethics Review Committee of Shandong Cancer Hospital (approval No. SDTHEC2022002002).

**Table 1:** Patient characteristics.

Characteristics	Value
Sex	
Male	7 (78%)
Female	2 (22%)
Age (years)	Median 73, range 65–89
Total PTV dose (Gy)	Median 50.4, range 41.4–60
Fraction	Median 28, range 23–30
Fraction dose	
1.8 Gy	2 (22%)
2.0 Gy	7 (78%)
TNM staging	
cT2N2M1	1 (11%)
cT3N2M0	2 (22.5%)
cT3N0M0	2 (22.5%)
cT4aN1M1	1 (11%)
pT2N0M0	1 (11%)
ypT3N0M0	1 (11%)
Uncertain	1 (11%)
Tumor location	
Cervical and upper thoracic esophagus	4 (45%)
Middle thoracic esophagus	4 (45%)
Lower thoracic esophagus	1 (10%)
Pathology biopsy	
Squamous cell carcinoma	9 (100%)
Adenocarcinoma	0

PTV, planning target volume. The clinical and histopathologic TNM (tumor node metastasis) classification stage was based on the UICC TNM 7th edition (19).

### 2.2 Image acquisition

Patients were placed in the supine position, and the head and neck were fixed with negative-pressure bags. The simulation CT images were obtained using a Philips large-aperture CT with a layer thickness of 3 mm. Next, an MRI simulator (Ingenia3.0T, Philips) was used to acquire localization MR images, including T1-weighted images (TR = 4.5 ms, TE = 2.0 ms, flip angle = 15°), T2-weighted images (TR = 7,059 ms, TE = 75 ms, flip angle = 110°) and T1-weighted enhanced images. The main role of the 3.0T MR images was to assist in delineating the gross tumor volume (GTV) and organs at risk (OARs) on the simulation CT images. Acquisitions were performed on an Elekta Unity MR-Linac system using T2W sequences (chest sequence parameters: TR = 2,100 ms, TE = 206 ms, SNR = 1, ACQ matrix M*P = 160*224) with a layer thickness of 1.2 mm before the treatment fraction started in stages. For each patient, the first eight treatment fractional MR images were collected. The primary role of the 1.5T MR images was to provide image guidance before the start of the treatment fraction.

### 2.3 Delineation

Delineation was performed with the simulation and daily 1.5T MR images. A specialist in EC radiotherapy delineated seven ROIs. The primary esophageal lesions and enlarged lymph nodes were delineated as the GTV based on CT and MR imaging. The clinical target volume (CTV) was defined as extension of the GTV up and down by a 3 cm margin and axially outward by a 0.5 cm margin as well as areas of lymphatic drainage corresponding to each segment of the esophagus. The planning target volume (PTV) was defined by expanding the CTV in this study by a 0.5 cm margin. The OARs, such as the body, lung (Lung-All/L/R), spinal cord, and heart, were further delineated. The delineation of ROIs was based on the guidelines for OARs in thoracic radiation therapy developed by the Radiation Therapy Oncology Group (RTOG), the European Organization for Research and Treatment of Cancer (EORTC), and Southwestern Oncology Group (SWOG) lung cancer committees (20).

### 2.4 Plan design

Using the Unity MR-Linac-specific TPS Monaco (v5.40.02) and a GPU-based Monte Carlo dose calculation platform (GPUMCD), reference plans were created for each patient while referencing the 1.5 T images. Nine patients were planned to use six to nine fields to generate intensity-modulated radiotherapy (IMRT) plans based on prescribed dose requirements. All plans required that the prescribed dose cover more than 95% of the target volume, and the maximum dose was not to exceed 110% of the prescribed dose. The OAR dose limit was based on International Commission on Radiation Units and Measurements (ICRU) Report No. 83 (see **Supplementary Table 1**).

### 2.5 Treatment implementation

**Figure 1:**
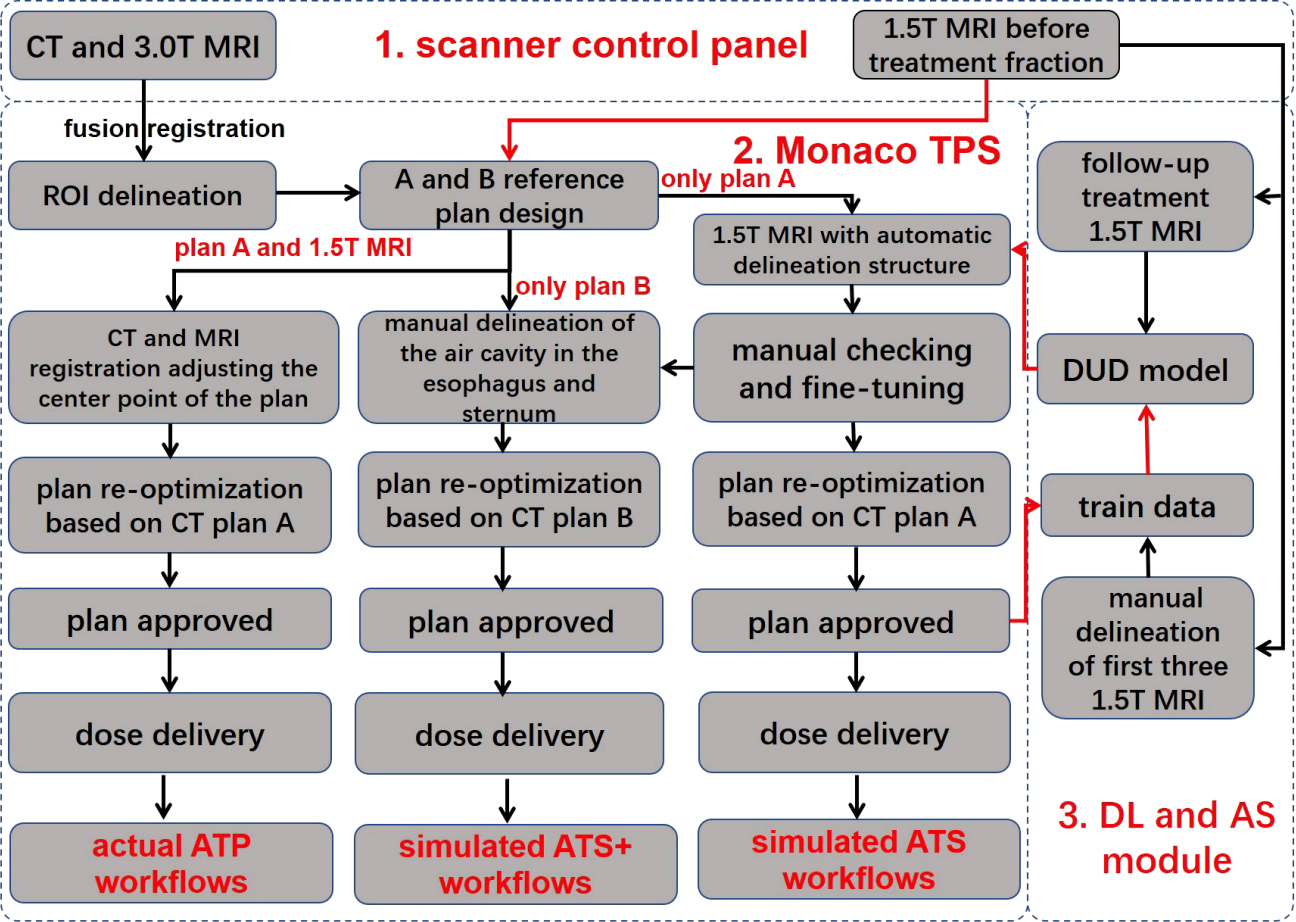
Workflow overview. The workflow consists of three main components, namely, the scanner control panel, the Monaco treatment planning system, and the deep learning and automatic segmentation module. The red line indicates automatic data delivery, and the black line indicates manual data transfer. TPS, treatment planning system; DUD model, daily updated delineation model; DL, deep learning; AS, automatic segmentation; ROI, region of interest; ATP, adapt to position; ATS, adapt to shape; ATS+, adapt to shape (including the newly added air cavity in the esophagus and the sternum). Reference plan (A) reference CT excludes the air cavity in the esophagus and the sternum; reference plan (B) reference CT includes the air cavity in the esophagus and the sternum.

**Figure 1** shows the three different treatment workflows used in this study.

#### 2.5.1 Actual adapt-to-position workflow

In the ATP radiation therapy mode based on position correction, online MR images could be registered with the planned CT to obtain movement parameters, correct the plan center point, and perform reoptimization based on reference plan A to obtain a new online treatment plan.

#### 2.5.2 Simulated adapt-to-shape workflow

**Figure 2:**
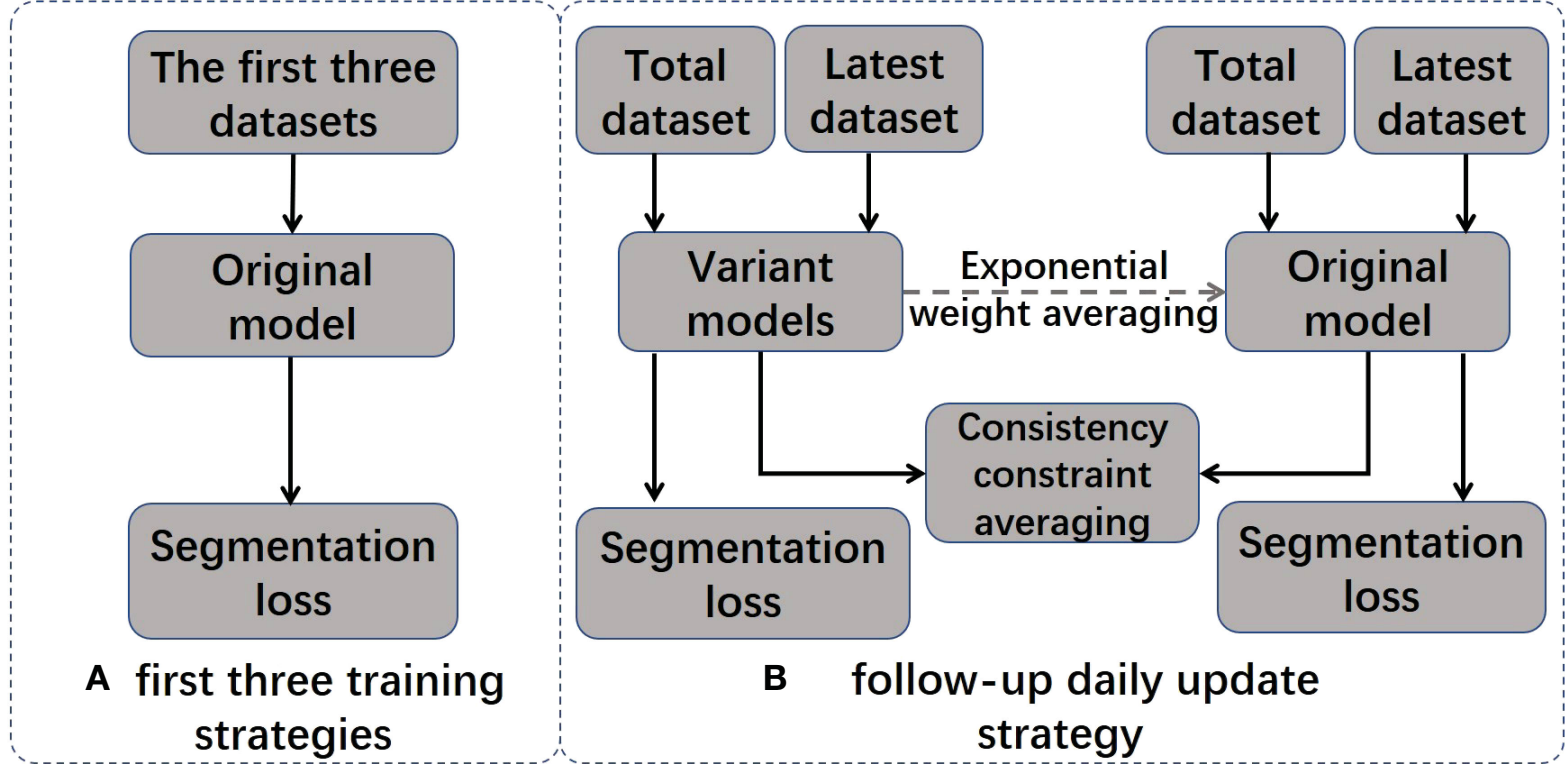
Two training strategies. The original model is included in the final model.

In the ATS radiation therapy mode based on shape correction, redelineation of the ROIs is required to optimize the new plan based on reference plan A. MR images and hand-delineated structures of the first three treatment fractions were selected as input data to predict the segmentation of the next fraction. The predicted segmentation structure was manually modified to deliver the treatment and was used as new input data to train the updated delineation model after the treatment ended, with a total of five predicted segments equating to five training updates of the model. Therefore, a total of (5 + 3) data training sessions and five prediction segmentations were performed for each patient. In addition, the training update of the model was arranged after treatment, between two fractions, and did not occupy the time of adaptive radiation therapy (ART). For the details of the training strategy and technical details of the network, see **Figure 2** and **Table 2**.

**Table 2:** Preprocessing techniques and hyperparameter settings.

		Value
1. Preprocessing techniques	Target spacings	(1.0, 1.0)
	Normalization	Cross-norm (22)
	Window crop size	(320, 320)
2. Data augmentation techniques (preprocessing techniques)	Scaling range	0.7–1.4
	Rotation range	−30°–30°
	Spatial transform prob.	0.9
	Gaussian noise prob.	0.3
	Gaussian kernel sigma	(0.25, 1.5)
	Gaussian blur prob.	0.3
	N segments of non-linear shift	5
	Non-linear shift prob.	0.5
3. Hyperparameter settings for training the model	Learning rate	3e-4
	Optimizer	Adam
	Weight decay	1e-4
	EWA ratio	0.99
	Batch size	16

EWA ratio, exponential weight averaging ratio.

#### 2.5.3 Simulated adapt-to-shape (+) workflow

First, ATP is based on the CT electron density for dose calculation, which considers the electron density of the air cavity in the esophagus and the sternum, and therefore, dosimetric errors are not produced. However, the ATS is the average electron density assignment to the ROIs in plan calculations. If the air cavity in the esophagus and the sternum are not individually contoured for assignment, then these two structures will be assigned values according to the CT-based average electron density of the body, inevitably resulting in dosimetric errors. Therefore, based on the above considerations, a simulated ATS+ workflow is implemented. A new CT reference plan was generated by manually adding the air cavity in the esophagus and sternum to the original reference CT with no change in the optimization constraints. To distinguish it from the original reference plan A, we define this plan as reference plan B. In parallel, the air cavity in the esophagus and the sternum were manually delineated based on MRI, and a new treatment plan was generated based on reference plan B by performing the simulated ATS workflow again.

### 2.6 Training strategy and technical details

#### 2.6.1 Training strategy

The model training strategy is visualized in **Figure 2**, and it includes two different training stages: initial training and daily updates. In the initial training stage, the original model is trained using MRI images and the manual contour results from the first three treatments of the current patient. In the daily update stage, a structure-aware regularization technique is introduced to further optimize the model. This strategy improves the accuracy of automatic segmentation models by jointly optimizing the weights of two models (original model *θ^original^* and variant model *θ^variant^*) and defining pointwise segmentation loss ${ℒ}_{\text{seg}}$ the consistency constraint ${ℒ}_{\text{cons }}$ . ${ℒ}_{\text{seg}}$ applies to the entire dataset, and ${ℒ}_{\text{cons }}$ only applies to the most recent dataset. We represent the segmentation result of the model as $p^{\left(i\right)}=\theta^{\left(i\right)}\left(x\right)$ , where x represents training images and $p^{\left(i\right)}\;$ presents the segmentation results for the i-th pixel in p ${ℒ}_{\text{seg}}$ and ${ℒ}_{\text{cons }}$ are denoted as follows:


$${ℒ}_{\text{seg}}={ℒ}_{ce}+{ℒ}_{\text{dice }}$$

$${ℒ}_{\text{cons}}={ℒ}_{vol}+{ℒ}_{len\;}+{ℒ}_{cen\;}$$

Specific formulas for the loss functions ${ℒ}_{ce},\;{ℒ}_{\text{dice }},\;{ℒ}_{vol},\;{ℒ}_{\text{len}},\text{ and }{ℒ}_{\text{cen}}$ can be found in **Appendix A**.

The joint loss function is presented as follows:


$${ℒ}_{\text{joint }}={ℒ}_{\text{seg}}+{ℒ}_{\text{cons }}$$

The joint loss function ${ℒ}_{\text{joint }}$ is only applied to the variant model to optimize its performance. After each daily update, the weights in the original model are gradually inherited from consecutive variant models using exponential weight averaging at a value of β = 0.99 to obtain the final model $\theta_{\text{original}}^{'}$ .


$$\theta_{\text{original}}^{'}=\beta \cdot \theta_{\text{original}}+\left(1-\beta \right)\cdot \theta_{\text{variant}}$$

#### 2.6.2 Details of the network and training strategy

The network structure used in this study was modified from nnU-Net 2D, and instance normalization was replaced with cross normalization (21) to improve model performance and robustness. Additionally, two deep learning model training methods were used to achieve more accurate and reliable tumor segmentation. Prior to training, the same preprocessing approach was applied to the datasets used for both training methods using the standard processing flow recommended for nnU-Net (15). In addition to the default data augmentation approach proposed for nnU-Net, Gibbs noise (22) and k-space spikes (23) were randomly superimposed on the image for data augmentation. The enhanced data were then imported into the training model. During training, the engine randomly selected a pair of preprocessed samples from both the entire dataset and the latest dataset in each iteration for optimizing seg and cons. Detailed information on the preprocessing techniques and hyperparameter settings can be found in **Table 2**.

### 2.7 Evaluation

#### 2.7.1 Delineation time

In the ATS workflow, the mean/range of traditional contouring (TC) time and actual contouring (AC) time were counted and compared, where the AC included automatic delineation (AD) and manual modification (MD). Additionally, the daily model training time was evaluated.

#### 2.7.2 Segmentation evaluation metrics

To confirm the reliability of the autodelineation model, the autodelineation results and the manual delineation results of the first eight iterations were compared and evaluated. In addition, to verify the optimization and upgrading effect of the automatic delineation model, MRI 8 (the 1.5 T MR images acquired before the eighth treatment) was automatically delineated by Models 1, 2, 4, 6, and 8, and the results were compared with the manual delineation results.

To evaluate the AS performance of the daily models, we used five quantitative metrics, including the Dice similarity coefficient (DSC), Hausdorff distance (HD), average symmetric surface distance (ASSD), maximum symmetric surface distance (MSSD), and relative area/volume difference (RAVD), to analyze the results in terms of overlapping and volumetric and spatial variations. Higher DSC values and lower HD, ASSD and MSSD, and RAVD values indicated more accurate segmentation results.

DSC measures the volumetric overlap of two sets of data and was obtained with the following equation:


(1) $$DSC\left(A,B\right)=\frac{2\left|A{\;}^{\cap }B\right|}{\left|A\right|+\left|B\right|}$$

A DSC of 1 means a perfect segmentation, whereas a DSC of 0 means no overlap at all.

Furthermore, the 95th percentile of the Hausdorff distance (HD95) was used to describe the surface data (24).


(2) $$h_{95}\left(A,B\right)={\;}^{95}K_{a\in A}^{th}\underset{b\in B}{min}∥b-a∥$$

The ASSD and MSSD are defined as follows:


(3) $$\text{ASSD}=\frac{1}{\left|A\right|+\left|B\right|}\left(\sum_{a\in Ab\in B}^{\;min}\|a-b\|+\sum_{b\in Ba\in A}^{\;min}\|b-a\|\right)$$

(4) $$\text{MSSD}=max\left(\underset{a\in A}{\max}\underset{b\in B}{\min}∥a-b∥,\underset{b\in A}{\max}\underset{a\in B}{\min}∥b-a∥\right)$$

where A and B indicate the boundary points on the automatically segmented set and ground-truth set, respectively.

The RAVD is defined as follows:


(5) $$\text{RAVD}=\frac{FP-FN}{TP+FN}$$

where TP, FP, and FN refer to true-positive predictions, false-positive predictions, and false-negative predictions, respectively (25).

#### 2.7.3 Plan evaluation

Since some patients’ tumors were located in the cervical or upper thoracic esophagus, the proportion of the heart volume receiving a dose of 5 Gy or above to the total volume was zero; thus, the patients were divided into two groups: A and B. Group A had data only on the heart mean dose (MHD), and group B had heart dose–volume histogram (DVH) parameters for all statistics. Compared to the ATP plan, the ATS plan corrects the deformation of the esophagus; thus, the ATS plan was evaluated in comparison to the ATP plan. In addition, since the ATS plan is based on average electron density assignment for ROIs and then the plan is optimized, the ATS plan and ATS+ plan (including the newly added air cavity in the esophagus and the sternum) were compared.

#### 2.7.4 Target dose assessment indicators

These variables included the target dose conformity index (CI), heterogeneity index (HI), average dose (D_mean_), D2%, D50%, D98%, and D100%. The CI was calculated as $CI=\frac{TV1*TV1}{TV*VR1}$ , where TV1 is the target volume that receives the prescription dose, TV is the target volume, and VR1 is the total volume of the prescription isodose. When a reference isodose entirely encircles the PTV without reaching the surrounding tissue, CI = 1, indicating that a hypothetically perfect conformal treatment has been accomplished (26). The HI was calculated as $HI=\frac{\text{ Dose that covers }x\backslash \%\text{ of tissue }\left(x=\text{High Dose Ref.}\backslash \%\right)}{\text{ Dose that covers }y\backslash \%\text{ of tissue }\left(y=\text{ Min. Dose Ref.}\backslash \%\right)}$ , where x = D2% and y = D98%. The heterogeneity of the dosage distribution will increase the closer HI is to zero.

### 2.8 Organ-at-risk assessment indicators

These variables included the mean whole lung dose (MLD), V5, V10, V20, V30, and V40 (Vx: proportion of the target organ receiving x Gy or more to total volume); the mean heart dose (MHD) and corresponding V5, V10, V20, V30, and V40; and the spinal cord D_max_.

### 2.9 Statistical analysis

Statistical analysis of dosimetric differences in the target areas and OAR between the ATS and ATP plans was performed with SPSS version 25.0 (IBM Corporation). A non-parametric test was used for comparisons between groups; P< 0.05 indicated a statistically significant difference, and values are expressed as the mean ± SD.

## 3 Results

### 3.1 Delineation time

As shown in **Supplementary Figure 1**, the mean/range of time spent on AC and TC using the current workflow was 5.54/2.28–13.58 min and 28.20/9.30–52.00 min, respectively. The AC time consisted of an AD mean/range of 1.40/1.10–1.78 min and an MD mean/range of 4.14/0.80–12.30 min. The time used for AC was substantially shorter than that needed for TC (P< 0.05). In addition, the mean/range of daily model training update time was 56/48–75 min.

### 3.2 Delineation accuracy

**Figure 3:**
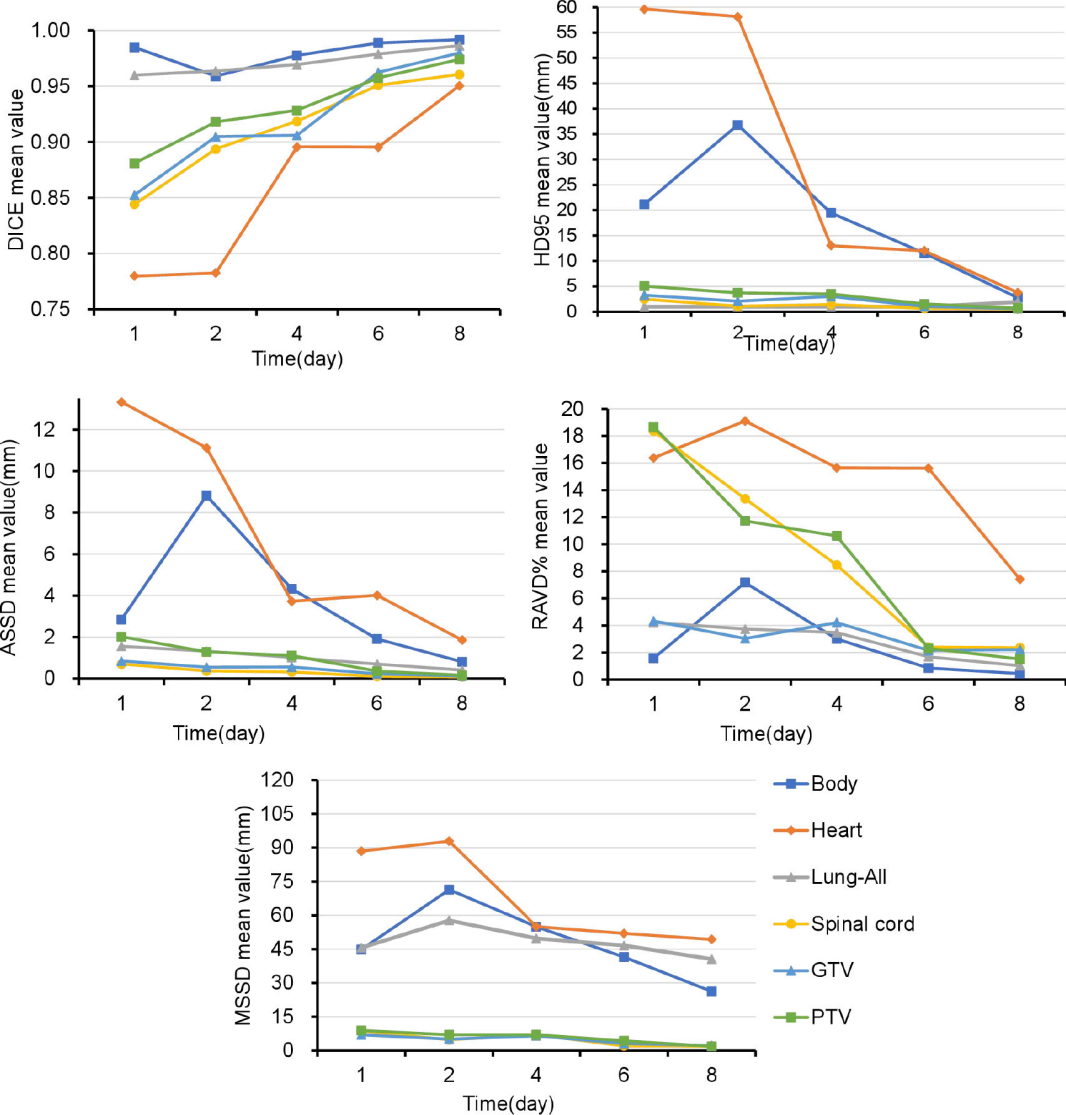
Over time, the Dice similarity coefficients continued to become closer to 1, and the 95th percentile of the Hausdorff distance, average symmetric surface distance, maximum symmetric surface distance, and relative area/volume difference values continued to decrease to 0. GTV, gross target volume; PTV, planning target volume; OARs, organs at risk; DSC, Dice similarity coefficient; HD, Hausdorff distance; ASSD, average symmetric surface distance; MSSD, maximum symmetric surface distance; RAVD, relative area/volume difference.

**Figure 3** and **Supplementary Table 2** show that the DSC continued to grow closer to 1; the HD95, ASSD, MSSD, and RAVD values continuously decreased toward 0; and the delineation accuracy for the OARs, GTV, and PTV improved with increasing numbers of fractions. After two training sessions for the deep learning module, the DSCs of all ROIs were greater than 0.90 except for those of the heart (DSC 0.78) and spinal cord (DSC 0.89); after four training sessions, the DSCs of all ROIs were greater than 0.90; and after eight training sessions, the DSCs of all ROIs were greater than 0.95. **Table 3** shows that the mean ± SD GTV DSC of the first eight automatic contours was 0.96 ± 0.03, HD95 was 1.66 ± 0.96 mm, ASSD was 0.31 ± 0.19 mm, MSSD was 5.40 ± 2.33 mm, and RAVD was 2.91 ± 1.99%, and the target area reflected excellent consistency. However, the mean ± SD heart DSC for the first eight automatic contours was 0.94 ± 0.09, which was less consistent than that of the other OAR contours.

**Table 3:** Parameters for evaluating delineation differences.

ROIs	Model with online updates: mean ± SD of first eight values				
	DSC	HD95 (mm)	ASSD (mm)	MSSD (mm)	RAVD (%)
GTV	0.96 ± 0.03	1.66 ± 0.96	0.31 ± 0.19	5.40 ± 2.33	2.91 ± 1.99
PTV	0.93 ± 0.05	2.89 ± 3.04	0.87 ± 0.87	6.99 ± 5.80	10.93 ± 14.96
Body	0.98 ± 0.03	16.43 ± 23.71	3.91 ± 5.71	53.97 ± 36.95	2.61 ± 4.83
Lung-All	0.98 ± 0.01	3.25 ± 3.56	0.70 ± 0.74	25.61 ± 38.56	2.00 ± 1.76
Lung-L	0.98 ± 0.02	2.80 ± 3.27	0.51 ± 0.60	15.84 ± 35.93	1.82 ± 2.76
Lung-R	0.98 ± 0.01	3.22 ± 3.97	0.71 ± 1.07	24.25 ± 42.76	1.41 ± 1.76
Heart	0.94 ± 0.09	22.51 ± 49.15	4.24 ± 8.84	45.02 ± 72.84	13.34 ± 26.56
Spinal cord	0.95 ± 0.04	1.38 ± 2.83	0.25 ± 0.42	4.77 ± 6.43	6.53 ± 6.78

ROIs, regions of interest; GTV, gross target volume; PTV, planning target volume; OAR, organ at risk; DSC, Dice similarity coefficient; HD, Hausdorff distance; ASSD, average symmetric surface distance; MSSD, maximum symmetric surface distance; RAVD, relative area/volume difference.

### 3.3 Adapt-to-position and adapt-to-shape plan comparison

#### 3.3.1 Group A dosimetric parameters

**Figure 4:**
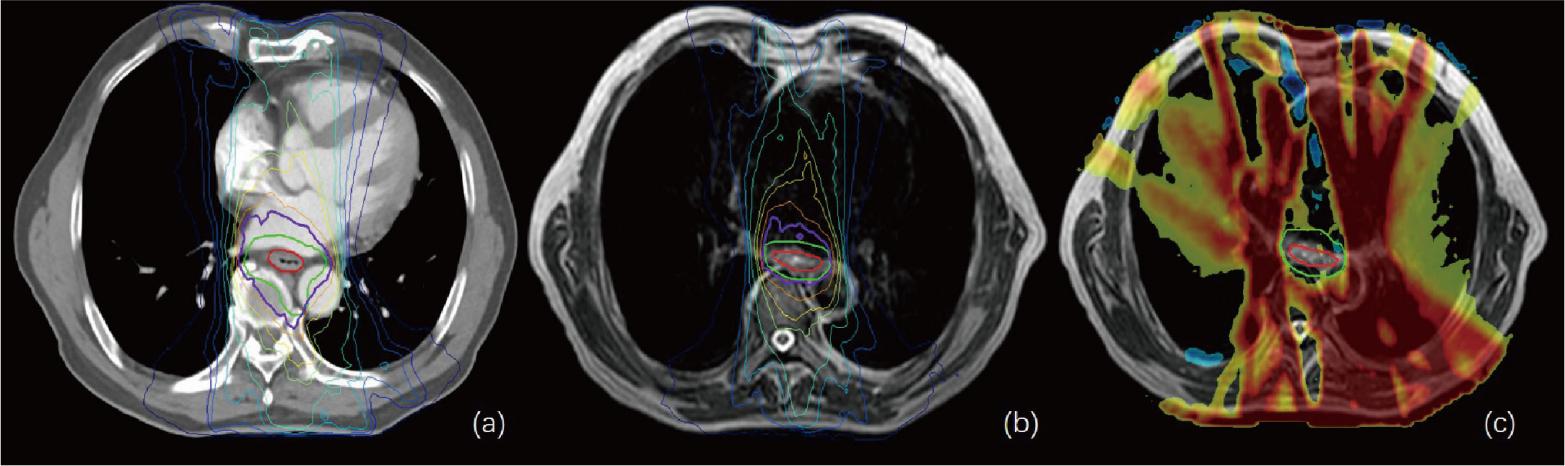
**(A, B)** show the isodose curves of the adapt-to-position (ATP) and adapt-to-shape (ATS) plans, respectively. **(C)** indicates the ATP dose minus the ATS dose. The red line represents the gross target volume (GTV), and the green line represents the planning target (PTV). This figure shows that the OAR lung and heart doses in the ATS plan were significantly smaller than those in the ATP plan, while the target–volume PTV dose was similar or slightly increased.

As shown in **Table 4** and **Figure 4**, the ATS plan demonstrated a lower HI (1.06 ± 0.03) than the ATP plan [HI (1.10 ± 0.02)] (P< 0.05). The D98 and D100 values of the ATS plan were greater than those of the ATP plan (P< 0.05). Obviously, the PTV coverage and the uniformity of the dose distribution of the ATS plan were better than those of the ATP plan. The V20 and V30 (17.27% ± 5.76% and 2.89% ± 0.82%) of the lungs in the ATS plan were significantly below those in the ATP plan (18.71% ± 5.04% and 9.88% ± 1.68%) (P< 0.05) by 23.38% and 40.17%, respectively. When compared with those in the ATP plan, the mean dose for the lung and heart in the ATS plan declined by 18.04% and 13.05%, respectively (p< 0.05). The exposed dose to the lungs and heart was significantly lower.

**Table 4:** Dosimetric parameter comparison.

DVH parameter		ATP	ATS	Reduction (%)	P value
		Mean ± SD	Mean ± SD		
Lung	V5 (%)	24.26 ± 5.82	22.54 ± 6.58	7.09	0.007
	V10 (%)	18.71 ± 5.04	17.27 ± 5.76	7.70	0.012
	V20 (%)	9.88 ± 1.68	7.57 ± 1.89	23.38	0.000
	V30 (%)	4.83 ± 0.95	2.89 ± 0.82	40.17	0.000
	V40 (%)	2.43 ± 0.77	1.22 ± 0.49	49.79	0.000
	D_mean_ (cGy)	598.54 ± 103.00	490.54 ± 113.15	18.04	0.000
Heart	D_mean_ (cGy)	111.98 ± 32.17	97.37 ± 59.37	13.05	0.003
PTV	D2 (cGy)	5,765.78 ± 498.30	5,675.94 ± 466.70	1.56	0.001
	D50 (cGy)	5,556.35 ± 470.55	5,538.57 ± 468.09	0.32	NS
	D98 (cGy)	5,258.35 ± 464.55	5,345.77 ± 515.37	-1.66	0.000
	D100 (cGy)	4,148.38 ± 415.55	4,979.33 ± 561.74	-20.03	0.000
	D_mean_ (cGy)	5,527.77 ± 474.86	5,492.88 ± 480.76	0.63	0.018
Spinal cord	D_max_ (cGy)	4,419.53 ± 662.71	3,897.43 ± 625.36	11.81	0.000
HI		1.10 ± 0.02	1.06 ± 0.03	3.64	0.000
CI		0.76 ± 0.03	0.77 ± 0.03	-1.32	NS

Reduction (%) = (ATP − ATS)/ATP*100. GTV, gross target volume; PTV, planning target volume. CI, conformity index; HI, heterogeneity index. NS, not signiﬁcant (P > 0.05).

#### 3.3.2 Group B dosimetric parameters

**Figure 5:**
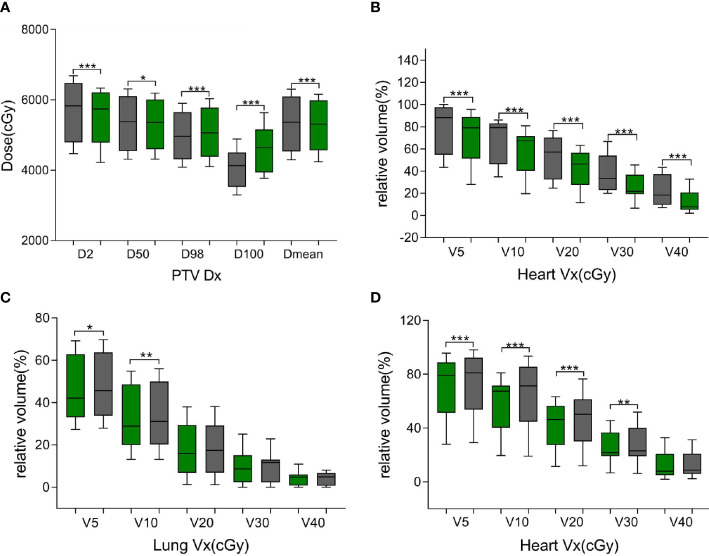
Green bars indicate the ATS plan dose–volume histogram (DVH) parameters, and gray bars indicate the ATP plan DVH parameters in **(A, B)** and the ATS+ plan DVH parameters in **(C, D)**. **(A)** denotes the dose at X% of the PTV and the average dose of the PTV. **(B, D)** denote the relative volume of the heart dose for Vx. **(C)** denotes the relative volume of the lung dose for Vx. * represents P< 0.05, ** represents P< 0.01, and *** represents P< 0.001.

As shown in **Supplementary Table 3** and **Figures 5A, B**, the same trend of variation in dosimetric parameters (except for the heart) was observed, and all dosimetric parameters of the heart were smaller in the ATS plan than in the ATP plan, especially in V20 and V30 (41.87% ± 15.78% and 25.31% ± 11.19% in the ATS plan vs. 53.71% ± 18.45% and 37.43% ± 16.28% in the ATP plan) (P< 0.05), which decreased by 22.04% and 32.38%, respectively. Similarly, the mean dose in the heart of the ATS plan decreased by 22.69% relative to that in the ATP plan (p< 0.05).

### 3.4 Daily adapt-to-shape+ and adapt-to-shape plan comparison

As shown in **Table 5**, in comparison with the ATS+ plan, the ATS plan displayed a smaller HI (1.06 ± 0.03 vs. 1.07 ± 0.02) (P< 0.05). In the lung tissue, V5 and V10 (26.74% ± 7.77%, 20.86% ± 6.44%) of the ATS+ plan were significantly higher (P< 0.05) than those of the ATS plan (22.54% ± 6.58%, 17.27% ± 5.76%) by 15.71% and 17.21%, respectively. The mean lung dose of the ATS+ plan was 14.26% greater than that of the ATS plan (p< 0.05). Therefore, the exposure dose in the low-dose region of the lungs was underestimated when the sternal and esophageal cavities were not considered. As shown in **Supplementary Table 4** and **Figures 5C, D**, the dosimetric changes in V5 and V10 in the lung tissue were similar to those described earlier. However, V5, V10, V20, V30, and D_mean_ were increased by 3.45%, 11.70%, 10.71%, 7.59%, and 8.24%, respectively, in the heart in the ATS+ plan relative to the ATS plan (P< 0.05), while V40 was not statistically distinct between the two plans. Apparently, the exposure dose to the heart had also been underestimated.

**Table 5:** Dosimetric parameter comparison.

DVH parameter		ATS+	ATS	Reduction (%)	P value
		Mean ± SD	Mean ± SD		
Lung	V5 (%)	26.74 ± 7.77	22.54 ± 6.58	15.71	0.005
	V10 (%)	20.86 ± 6.44	17.27 ± 5.76	17.21	0.004
	V20 (%)	8.69 ± 2.07	7.57 ± 1.89	12.89	NS
	V30 (%)	3.18 ± 0.83	2.89 ± 0.82	9.12	NS
	V40 (%)	1.36 ± 0.51	1.22 ± 0.49	10.29	NS
	D_mean_ (cGy)	572.11 ± 141.07	490.54 ± 113.15	14.26	0.003
Heart	D_mean_ (cGy)	103.69 ± 61.59	97.37 ± 59.37	6.10	NS
PTV	D2 (cGy)	5,703.96 ± 468.74	5,675.94 ± 466.70	0.49	0.009
	D50 (cGy)	5,542.55 ± 475.90	5,538.57 ± 468.09	0.07	NS
	D98 (cGy)	5,349.05 ± 494.06	5,345.77 ± 515.37	0.06	NS
	D100 (cGy)	5,038.88 ± 487.54	4,979.33 ± 561.74	1.18	NS
	D_mean_ (cGy)	5,496.40 ± 483.65	5,492.88 ± 480.76	0.06	NS
Spinal cord	D_max_ (cGy)	3,811.49 ± 503.77	3,897.43 ± 625.36	-2.25	0.049
HI		1.07 ± 0.02	1.06 ± 0.03	0.93	0.015
CI		0.77 ± 0.05	0.77 ± 0.03	0.00	NS

Reduction (%) = [ATS(+)-ATS]/ATS(+)*100. ATS, adapt to shape; ATS+, adapt to shape (including the newly added air cavity in the esophagus and the sternum); GTV, gross target volume; PTV, planning target volume. CI, conformity index; HI, heterogeneity index. NS, not signiﬁcant (P > 0.05).

## 4 Discussion

Numerous recent studies have counted the time to target volume and OAR redelineation time during online ART. Stanescu et al. (27), in a study of MRgoART stereotactic body radiation therapy (SBRT) for abdominal tumors on MR-Linac, found that the mean/range of time required for image registration and delineation of liver and pancreatic tumors was 14.4/5–34 min and 14.6/7–28 min, respectively. Similarly, Daamen et al. (28), in a study of MRgoART SBRT for unresectable malignancies in the upper abdomen using MR-Linac, determined that the median/range of time required for redelineation was 13/3–38 min. In our study, the mean/range of time used for AC with the existing workflow was 5.54/2.28–13.58 min. In addition, the mean/range of daily model training update time reached up to 56/48–75 min, but it did not increase the time of the online ATS workflow, which was scheduled after the final completed treatment fraction of the day. Compared to traditional delineation, the automatic delineation model we introduced required significantly less time to redelineate the ROIs. Furthermore, the reduction in delineation time could decrease the incidence of postural shift in patients during the ATS workflow, thus enhancing the accuracy of dose delivery. This allowed the ATS workflow to achieve a similar speed to the ATP workflow while maintaining the dosimetric advantage.

A large number of researchers have undertaken numerous studies on automatic image segmentation by deep learning; however, the majority of available automatic segmentation measures are based on the deep learning of large samples. For instance, Tang et al. (11) proposed a convolutional neural network for liver segmentation and included 282 datasets, achieving a median DSC of 0.94. At least 100 patients with CT or MR images needed to be enrolled to train the model, which would take a great deal of time. In addition, similar high-quality images are not easily collected, and the reproduction of experimental results is difficult. This implies that there are obstacles to the application of deep learning models with large samples in ATS workflows. Therefore, there is a strong need for research based on small-sample deep learning networks, and Chen et al. (29) propose a personalized AS framework to assist in the online delineation of prostate cancer using MRgoART. The study used only first-fraction images, and contour data from 16 patients were used to train the population AS model. The mean DSC of all ROIs in the test set was greater than 0.92. The feasibility of applying MRgoART to abdominal tumors using small-sample deep learning networks was demonstrated. Similarly, in our study, we used only a small amount of data to train an automatic patient-specific deep learning–based delineation model and embedded it in the ATS workflow, which was updated in the time between radiation treatment fractions. The average DSC of all ROIs for EC in our proposed approach exceeded 0.9 after only four rounds of training. We also found that the automatic cardiac segmentation effect was not ideal compared to that for other OARs, reaching a DSC value of only 0.78 after two training sessions, while the DSC values of other OARs were greater than 0.89. This was mainly because the heart is always beating, and the volume of different treatment fractions varied greatly. However, the cardiac segmentation effect improved significantly as the number of training sessions increased, and the DSC value reached 0.9 after four training sessions. Such a segmentation effect with minor modifications fully met the thoracic tumor treatment needs of the ATS workflow. Furthermore, the timely update of the segmentation model allowed it to better adapt to the daily changes of a specific patient, which is not possible with other deep learning models.

In terms of dosimetry, D98 and D100 were greater in the ATS plan than in the ATP plan, and the HI was lower (P< 0.05). Obviously, the target volume coverage and the uniformity of the dose distribution of the ATS plan were better than those of the ATP plan, which is consistent with the previous findings of Winkel et al. (8). Likewise, the ATS plan showed clear benefits in terms of OAR dose reduction. In our study, the relative mean cardiac dose in patients with cervical and upper thoracic esophageal cancer in the ATS plan decreased by 13.05% (P< 0.05) relative to the ATP plan, with a dose of only 0.15 Gy. However, due to the proximity of the heart to middle thoracic and lower thoracic esophageal cancer tumors, the volume and dose of irradiation were larger, and the relative mean dose decreased by 22.69% (P< 0.05) or 5.38 Gy. The relative mean dose to the lungs was less affected by the location of the esophageal cancer, decreasing by 18.04% (cervical and upper thoracic) and 12.36% (middle thoracic and lower thoracic) in the ATS plan, or 0.15 and 0.11 Gy, respectively. The finding is similar to that of Boekhoff et al. (30) in their MRgRT study of esophageal cancer, in which the average mean lung dose was reduced by 26%, and the average mean heart dose was reduced by 12%. In summary, as expected, the plan quality of the ATS workflow exceeded that of the ATP workflow since the ATS plan was equivalent to complete replanning. On the other hand, the ATS+ V5 and V10 were significantly higher in normal lung tissue (P< 0.05), and similarly, in the heart, V5, V10, V20, and D_mean_ were markedly increased (P< 0.05) (see **Figures 5C, D**, **Supplementary Table 4**). This indicated that we seriously underestimated the amount of OARs and lung and heart exposure in the low-dose region during ART for EC without contouring the sternum and the air cavity in the esophagus. This could result in an increased probability of radiotherapy-induced complications such as radiation pneumonia, constrictive pericarditis, and cardiac arrhythmias.

We also acknowledge the serious limitations of this study. First, the patients in this study included only those with EC from a single center. Second, ROIs with large volume changes caused by high-amplitude movement between treatment fractions, e.g., the heart, were not well delineated in the first few training sessions. Third, the approach introduced in this research was also trained to delineate only MR images derived from a 1.5 T MR-Linac system. The stability of the scanned data attained with other diverse modalities was not validated.

## 5 Conclusion

The accuracy and speed of AI-based AS in the ATS workflow met the clinical radiation therapy needs for EC. This allowed the ATS workflow to achieve a similar speed to the ATP workflow while maintaining the initially planned dose advantage. Fast and precise online ATS treatment ensured an adequate dose to the PTV while reducing the dose to the heart and lungs, thus reducing the toxic side effects caused by radiation therapy. Additionally, without delineating the structures of the air cavity in the esophagus and the sternum, the exposure doses to the lungs and heart were underestimated. This study can provide guidance in diminishing pulmonary and cardiac radiotherapy toxicities in the course of MRgoART for EC.

## Data availability statement

The raw data supporting the conclusions of this article will be made available by the authors, without undue reservation.

## Ethics statement

The studies involving human participants were reviewed and approved by the Ethics Review Committee of Shandong Cancer Hospital. Written informed consent for participation was not required for this study in accordance with the national legislation and the institutional requirements.

## Author contributions

Conceptualization: HW and ZL. Investigation, data acquisition, and analysis: HW, XL, YS, PY, JZ and XS. Original draft preparation: HW. Review and editing: ZL and YY. Supervision: YY. All authors contributed to the article and approved the submitted version.
