# Supplementary material for: Feasibility study of adaptive radiotherapy for esophageal cancer using artificial intelligence autosegmentation based on MR-Linac
Source: Front Oncol. 2023 Jun 8;13:1172135. doi: 10.3389/fonc.2023.1172135 (PMC10289262; doi:10.3389/fonc.2023.1172135)
Supplement: Supplementary file 1 [file DataSheet_1.doc]

**Appendix A.**

Given a structure y corresponding to
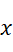
,
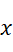
 represents training images.


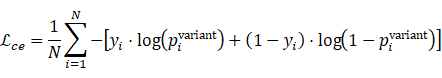


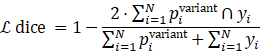


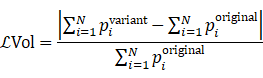


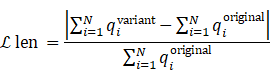


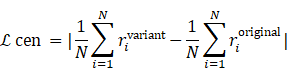


**Supplementary Table 1.** Dose constraints for the patients

| ROIs |  | Dose constraints |
| --- | --- | --- |
| PTV |  | V95% ≥ 100% of the dose prescribed |
|  |  | D_max_<110% of the dose prescribed |
| OAR | Lung – All | 14 Gy≤D_mean_≤16 Gy, V5≤60%, V20＝30%, V30≤20%  Patients with concurrent chemoradiotherapy V20≤28% |
|  | Heart | V30≤40%, V40≤30% |
|  | Spinal cord | D_max_≤45 Gy |

Abbreviations: ROIs, region of interest; OAR, organs at risk; PTV, planning target volume; D_mean_, mean dose; D_max_, maximum dose.

**Supplementary Table 2.** Parameters for evaluating delineation differences

| ROIs | Parameters | Value (Mean ± SD) | | | | |
| --- | --- | --- | --- | --- | --- | --- |
|  |  | MR day 1 | MR day 2 | MR day 4 | MR day 6 | MR day 8 |
| GTV | DSC | 0.80±0.07 | 0.92±0.02 | 0.93±0.02 | 0.96±0.01 | 0.97±0.01 |
|  | HD95 (mm) | 3.20±1.88 | 2.05±1.07 | 3.00±2.18 | 1.05±0.69 | 0.60±0.42 |
|  | ASSD (mm) | 0.80±0.50 | 0.56±0.22 | 0.56±0.31 | 0.23±0.11 | 0.13±0.10 |
|  | MSSD (mm) | 6.90±2.80 | 5.09±2.98 | 6.58±3.39 | 3.14±2.11 | 2.25±1.70 |
|  | RAVD% | 4.30±3.59 | 3.03±3.15 | 4.22±3.45 | 2.16±2.09 | 2.23±1.57 |
| PTV | DSC | 0.80±0.11 | 0.90±0.08 | 0.91±0.03 | 0.96±0.02 | 0.98±0.01 |
|  | HD95 (mm) | 5.00±2.45 | 3.70±2.60 | 3.49±1.28 | 1.53±0.90 | 0.69±0.42 |
|  | ASSD (mm) | 2.00±1.76 | 1.29±1.38 | 1.11±0.43 | 0.36±0.21 | 0.16±0.11 |
|  | MSSD (mm) | 8.90±3.24 | 7.04±3.32 | 7.03±2.11 | 4.34±2.85 | 1.87±0.81 |
|  | RAVD% | 18.68±16.45 | 11.73±14.29 | 10.61±8.92 | 2.31±2.27 | 1.51±0.75 |
| Body | DSC | 0.90±0.00 | 0.96±0.04 | 0.98±0.03 | 0.99±0.00 | 0.99±0.00 |
|  | HD95 (mm) | 21.20±26.65 | 36.82±36.42 | 19.46±29.11 | 11.54±18.96 | 2.75±0.90 |
|  | ASSD (mm) | 2.80±2.80 | 8.82±10.06 | 4.33±7.31 | 1.92±2.20 | 0.82±0.54 |
|  | MSSD (mm) | 44.87±36.51 | 71.41±43.77 | 54.89±41.15 | 41.50±38.49 | 26.23±29.31 |
|  | RAVD% | 1.50±0.96 | 7.17±9.46 | 3.01±5.94 | 0.86±0.68 | 0.44±0.28 |
| Lung-All | DSC | 0.9±0.01 | 0.96±0.01 | 0.97±0.01 | 0.98±0.01 | 0.99±0.00 |
|  | HD95 (mm) | 7.0±5.35 | 5.93±4.62 | 5.37±4.57 | 4.02±4.67 | 1.89±1.46 |
|  | ASSD (mm) | 1.5±1.08 | 1.33±0.55 | 1.02±0.56 | 0.71±0.43 | 0.41±0.22 |
|  | MSSD (mm) | 45.67±49.71 | 57.75±44.42 | 49.75±49.59 | 46.63±47.32 | 40.56±55.27 |
|  | RAVD% | 4.20±2.77 | 3.74±2.28 | 3.47±1.39 | 1.68±1.92 | 1.03±1.18 |
| Heart | DSC | 0.80±0.27 | 0.78±0.28 | 0.90±0.05 | 0.90±0.06 | 0.95±0.03 |
|  | HD95 (mm) | 59.64±81.81 | 58.15±79.18 | 13.03±8.09 | 12.00±6.24 | 3.77±2.34 |
|  | ASSD (mm) | 13.33±15.85 | 11.13±13.03 | 3.73±2.02 | 4.02±1.43 | 1.87±1.64 |
|  | MSSD (mm) | 88.46±92.94 | 92.90±99.16 | 55.01±70.74 | 116.30±95.92 | 49.34±73.45 |
|  | RAVD% | 16.38±11.58 | 19.10±11.89 | 15.65±7.22 | 15.62±9.56 | 7.41±5.30 |
| Spinal cord | DSC | 0.80±0.11 | 0.89±0.04 | 0.92±0.01 | 0.95±0.01 | 0.96±0.02 |
|  | HD95 (mm) | 2.40±2.86 | 1.07±0.50 | 1.43±0.98 | 0.63±0.11 | 0.51±0.24 |
|  | ASSD (mm) | 0.70±0.77 | 0.37±0.25 | 0.32±0.19 | 0.11±0.04 | 0.08±0.05 |
|  | MSSD (mm) | 8.70±7.41 | 4.76±4.09 | 7.12±6.22 | 2.04±0.93 | 1.94±0.88 |
|  | RAVD% | 18.36±17.60 | 13.37±9.95 | 8.47±3.34 | 2.41±1.97 | 2.36±1.81 |

Abbreviations: ROIs, region of interest; GTV, gross target volume; PTV, planning target volume; OAR, organs at risk; DSC, Dice similarity coefficient; HD, Hausdorff distance; ASSD, average symmetric surface distance; MSSD, maximum symmetric surface distance; RAVD, relative area/volume difference.

**Supplementary Table 3. Dosimetric parameter comparison**

| DVH parameter | | ATP | ATS | Reduction (%) | P value |
| --- | --- | --- | --- | --- | --- |
|  |  | Mean ± SD | Mean ± SD |  |  |
| Lung | V5 (%) | 50.61 ±10.89 | 46.43 ±14.56 | 8.26 | NS |
|  | V10 (%) | 35.57 ±10.95 | 32.35 ±14.58 | 9.05 | NS |
|  | V20 (%) | 21.09 ±9.61 | 17.47 ±12.24 | 17.16 | 0.007 |
|  | V30 (%) | 11.95 ±6.89 | 9.63 ±7.64 | 19.41 | 0.012 |
|  | V40 (%) | 6.02 ±1.04 | 4.42 ±3.03 | 26.58 | 0.008 |
|  | D_mean_ (cGy) | 1176.65 ±332.09 | 1031.24 ±430.33 | 12.36 | 0.004 |
| Heart | V5 (%) | 80.12 ±21.89 | 71.99 ±21.02 | 10.15 | 0.000 |
|  | V10 (%) | 69.87 ±20.56 | 58.25 ±18.33 | 16.63 | 0.000 |
|  | V20 (%) | 53.71 ±18.45 | 41.87 ±15.78 | 22.04 | 0.000 |
|  | V30 (%) | 37.43 ±16.28 | 25.31 ±11.19 | 32.38 | 0.000 |
|  | V40 (%) | 21.83 ±13.54 | 11.85 ±9.83 | 45.72 | 0.000 |
|  | D_mean_ (cGy) | 2370.52 ±727.06 | 1832.71 ±593.06 | 22.69 | 0.000 |
| PTV | D2 (cGy) | 5700.14 ±801.95 | 5547.82 ±705.97 | 2.67 | 0.000 |
|  | D50 (cGy) | 5341.23 ±721.98 | 5313.98 ±666.45 | 0.51 | 0.050 |
|  | D98 (cGy) | 4978.58 ±640.13 | 5070.34 ±668.73 | -1.84 | 0.000 |
|  | D100 (cGy) | 4090.19 ±499.76 | 4616.75 ±646.62 | -12.87 | 0.000 |
|  | D_mean_ (cGy) | 5330.34 ±722.85 | 5274.86 ±674.36 | 1.04 | 0.000 |
| Spinal cord | D_max_ (cGy) | 4673.66 ±601.07 | 4324.42 ±507.09 | 7.47 | 0.000 |
| HI |  | 1.14 ±0.04 | 1.10 ±0.05 | 3.51 | 0.000 |
| CI |  | 0.50 ±0.18 | 0.54 ±0.24 | -8.00 | NS |

Reduction (%) =(ATP-ATS)/ATP*100. Abbreviations: GTV, gross target volume; PTV, planning target volume. CI, conformity index; HI, heterogeneity index. NS, not signiﬁcant (P > 0.05).

**Supplementary Table 4. Dosimetric parameter comparison**

| DVH parameter | | ATS+ | ATS | Reduction (%) | P value |
| --- | --- | --- | --- | --- | --- |
|  |  | Mean ± SD | Mean ± SD |  |  |
| Lung | V5 (%) | 47.87±14.77 | 46.43 ±14.56 | 3.01 | 0.025 |
|  | V10 (%) | 33.28±14.92 | 32.35 ±14.58 | 2.79 | 0.001 |
|  | V20 (%) | 17.79±11.51 | 17.47 ±12.24 | 1.80 | NS |
|  | V30 (%) | 9.76±6.54 | 9.63 ±7.64 | 1.33 | NS |
|  | V40 (%) | 4.32±3.03 | 4.42 ±3.03 | -2.31 | NS |
|  | D_mean_(cGy) | 1054.13±409.38 | 1031.24 ±430.33 | 2.17 | NS |
| Heart | V5 (%) | 74.56±21.66 | 71.99 ±21.02 | 3.45 | 0.000 |
|  | V10 (%) | 65.97±23.14 | 58.25 ±18.33 | 11.70 | 0.000 |
|  | V20 (%) | 46.89±18.15 | 41.87 ±15.78 | 10.71 | 0.000 |
|  | V30 (%) | 27.39±12.89 | 25.31 ±11.19 | 7.59 | 0.002 |
|  | V40 (%) | 12.52±8.92 | 11.85 ±9.83 | 5.35 | NS |
|  | D_mean_(cGy) | 1997.30±683.92 | 1832.71 ±593.06 | 8.24 | 0.000 |
| PTV | D2 (cGy) | 5517.08±676.61 | 5547.82 ±705.97 | -0.56 | NS |
|  | D50 (cGy) | 5291.00±661.14 | 5313.98 ±666.45 | -0.43 | NS |
|  | D98 (cGy) | 5079.71±669.41 | 5070.34 ±668.73 | 0.18 | NS |
|  | D100 (cGy) | 4676.66±611.77 | 4616.75 ±646.62 | 1.28 | NS |
|  | D_mean_(cGy) | 5253.85±665.45 | 5274.86 ±674.36 | -0.40 | NS |
| Spinal cord | D_max_ (cGy) | 4300.00±483.16 | 4324.42 ±507.09 | -0.57 | NS |
| HI |  | 1.09±0.03 | 1.10 ±0.05 | -0.92 | NS |
| CI |  | 0.54±0.23 | 0.54 ±0.24 | 0.00 | NS |

Reduction (%)=[ATS(+)-ATS]/ATS(+)*100. Abbreviations: ATS, adapt-to-shape; ATS+, adapt-to-shape (including newly added esophageal cavity and sternum); GTV, gross target volume; PTV, planning target volume. CI, conformity index; HI, heterogeneity index. NS, not signiﬁcant (P > 0.05).


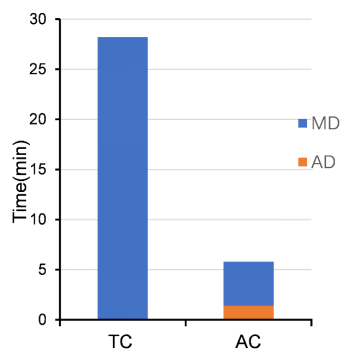


**Supplementary** **Fig. 1.** Traditional manual delineation time and actual delineation time of the existing workflow. MD indicates the time used for manual delineation or modification, and AD indicates the automatic delineation time.
